# Tail-vein injection of MSC-derived small extracellular vesicles facilitates the restoration of hippocampal neuronal morphology and function in APP / PS1 mice

**DOI:** 10.1038/s41420-021-00620-y

**Published:** 2021-09-04

**Authors:** Han Wang, Yuqi Liu, Junchen Li, Tian Wang, Yue Hei, Huiming Li, Xue Wang, Lina Wang, Ruijing Zhao, Weiping Liu, Qianfa Long

**Affiliations:** 1 Department of Neurosurgery, Xijing Hospital, Fourth Military Medical University, No. 127 Changle West Road, 710032 Xi’an, P.R. China; 2 Mini-invasive Neurosurgery and Translational Medical Center, Xi’an Central Hospital, Xi’an Jiaotong University, No. 161, West 5th Road, Xincheng District, 710003 Xi’an, P.R. China; 3 Affiliated Hospital of Yan’an University, Yongxiang Road, Baota District, 716000 Yan’an, China

**Keywords:** Neurological disorders, Regeneration and repair in the nervous system

## Abstract

Mesenchymal stem-cell-derived small extracellular vesicles (MSC-EVs), as a therapeutic agent, have shown great promise in the treatment of neurological diseases. To date, the neurorestorative effects and underlying mechanism of MSC-EVs in Alzheimer’s disease (AD) are not well known. Herein, we aimed to investigate the action of MSC-EVs on the neuronal deficits in β-amyloid protein (Aβ)-stimulated hippocampal neurons, or AD cell (SHSY5Y cell lines) and animal (APPswe / PS1dE9 mice) models. In the present study, the cell and AD models received a single-dose of MSC-EVs, and were then assessed for behavioral deficits, pathological changes, intracellular calcium transients, neuronal morphology alterations, or electrophysiological variations. Additionally, the nuclear factor E2-related factor 2 (Nrf2, a key mediator of neuronal injury in AD) signaling pathway was probed by western blotting in vitro and in vivo models of AD. Our results showed that MSC-EVs therapy improved the cognitive impairments and reduced the hippocampal Aβ aggregation and neuronal loss in AD mice. Markedly, EV treatment restored the calcium oscillations, dendritic spine alterations, action potential abnormalities, or mitochondrial changes in the hippocampus of AD models. Also, we found that the Nrf2 signaling pathway participated in the actions of MSC-EVs in the cell and animal models. Together, these data indicate that MS-EVs as promising nanotherapeutics for restoration of hippocampal neuronal morphology and function in APP / PS1 mice, further highlighting the clinical values of MSC-EVs in the treatment of AD.

## Introduction

Alzheimer’s disease (AD) is a neurodegenerative disorder characterized by a progressive decline in episodic memory as well as deficits in executive functioning, and is recognized by the World Health Organization as a global public health priority [1]. Currently, 60–80% of the total population suffering from dementia is ascribed to AD, and elderly individuals are considered to be more susceptible [2]. Previous studies have suggested that a cascade of neuropathological alterations occur in Alzheimer’s disease including amyloid plaque deposition, neuronal deficits, and glial activation [3, 4]. Specifically, β-amyloid (Aβ) is believed to contribute to the hippocampal neuron dysfunction that characterizes the stages of AD [5]. Although multiple treatment strategies have been used against pathological features in animal models and patients [6], immense challenge is to restore the neuronal structure and function in AD.

The development of regenerative medicine using stem cell therapy holds the promise in the treatment of neurological disorders [7]. In particular, mesenchymal stem cells (MSCs) exhibit a great therapeutic potential in AD owing to their biological characteristics, including low immunogenicity, anti-inflammatory properties, and the ease of isolation [8]. Accumulating evidences suggest that the small extracellular vesicles (EVs) secreted by MSCs are more effective as a restorative therapy in neurological injury than their parental cells [9]. Notably, EVs contain a large number of cargoes (e.g., RNA, protein, DNA, etc.) as well as have the ability to permeate the blood–brain barrier and are easy to use and preserve, making them favorable for development in basic and preclinical research [10]. Recent studies indicate that the MSC-EVs (or MSC-derived exosomes) can be selectively uptaken by neuronal cells as well as show neuroprotection and cognitive improvement in AD model [8, 11]. Additionally, our previous reports showed that MSC-EVs can target neurons and restore neurodegeneration following seizures [12]. As it’s known, interneuron dysfunction, including altered dendrite spines [3], impaired excitability [5], and intracellular calcium disruption [13] have emerged as potential mechanisms underlying the cognitive deficits in AD. Furthermore, accumulating data suggest that the neuronal injury is ascribed to oxidative damage besides neuroinflammation, which is also regarded as a key component in the pathogenesis of AD [14, 15]. Particularly, nuclear factor E2-related factor 2 (Nrf2) is responsible for regulating the oxidative stress and associated neuronal damage in multiple brain diseases [16, 17]. Thus, modulating these mechanisms may help to improve brain function in these conditions.

In this study, we aimed to develop MSC-EVs as cell-free nanotherapeutics to restore hippocampal structure and function in APP / PS1 transgenic mice. The results showed that EV therapy ameliorated Aβ aggregation, neuronal loss, and the cognitive deficits in AD mice, further repaired dendritic spine morphology, calcium transients, and action potentials of hippocampal neurons in cell or animal models. We also found that the Nrf2 signaling pathway participated in the oxidative defense produced by MSC-EVs, both in vitro and in vivo. Collectively, our results demonstrate the novel therapeutic effects of MSC-EVs in AD and shed light on the mechanism underlying its efficacy.

## Results

### MSC-EVs characterization and tracking

**Fig. 1:**
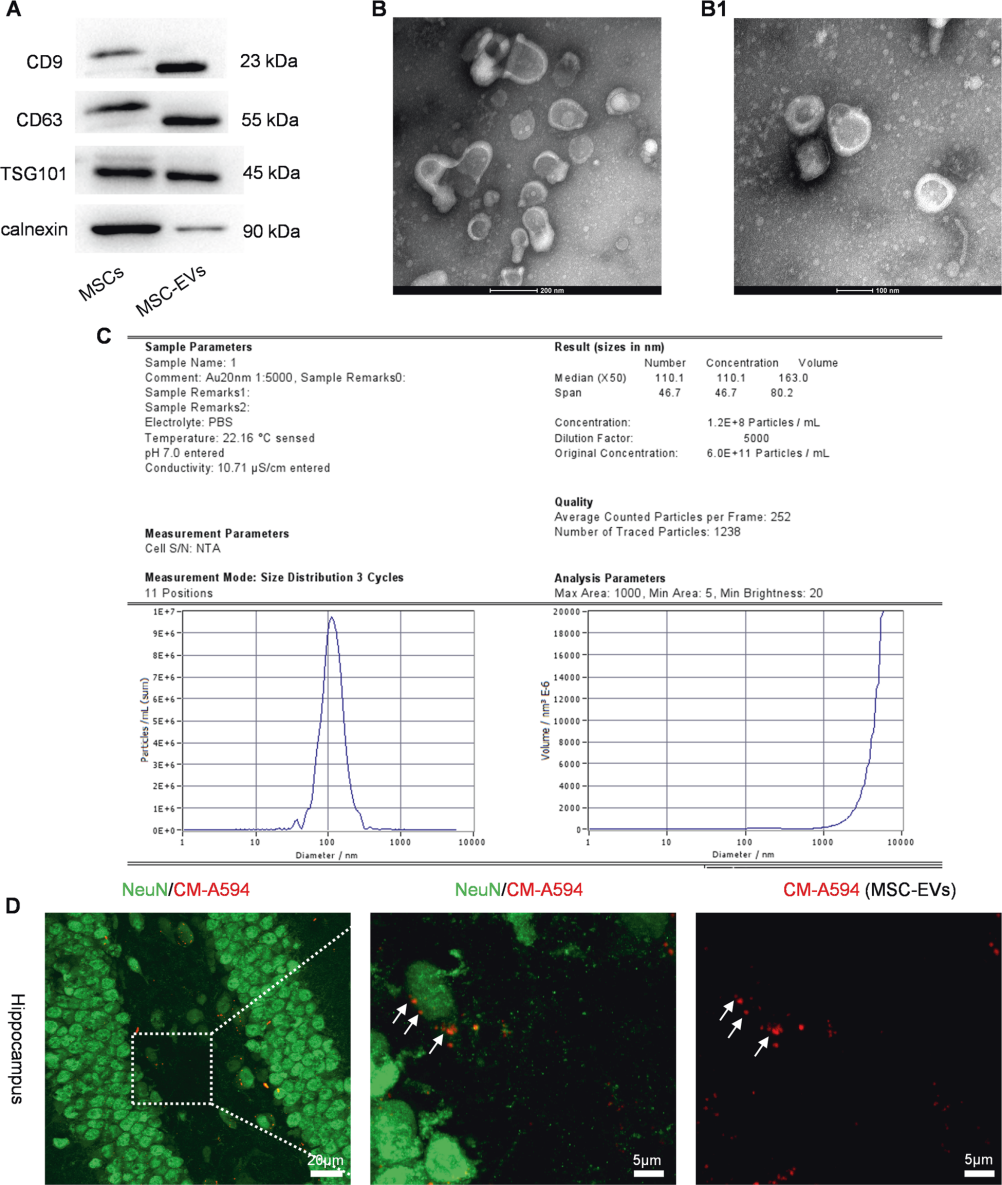
MSC-EVs characterization and tracking in APP / PS1 mice. **A** Western blots of CD9, CD63, TSG101 and calnexin in MSC-EVs and MSCs (control). **B**, **B1** transmission electron microscopy (TEM) images show the morphology of MSC-EVs (**B**, bar = 200 nm; **B1**, bar = 100 nm). **C** Nanoparticle tracking analysis (NTA) of size distribution for MSC-EVs. **D** Representative images show the location of C5 Maleimide-Alexa 594 (CM-A954) labeled MSC-EVs (red, white arrowheads) in the hippocampal neurons (green) in vivo (bar = 20 μm), the square area appears with higher magnification in the image on the right (bar = 5 μm).

Western blotting results showed that the EVs, isolated from the culture medium of MSCs, were positive for classical EV markers CD63, CD9, TSG101 found on the surface of MSCs and negative for calnexin (Fig. 1A). Additionally, TEM and NTA assay revealed that the morphology of EVs presented a typical cup-shape (Fig. 1B and B1) and major size distribution ranged from 40–160 nm (mean size = 110.1 ± 16.7 nm) in diameter (Fig. 1C) as previous reports [18]. After a tail vein injection of MSC-EVs for 24 h, we also found that CM-A594-labelled EVs were located in the cytoplasm of hippocampal neurons in APP / PS1 mice (Fig. 1D), suggesting the target of MSC-EVs within hippocampal neurons.

### Tail vein injection of MSC-EVs reduces hippocampal Aβ aggregation and neuronal loss in APP / PS1 mice

**Fig. 2:**
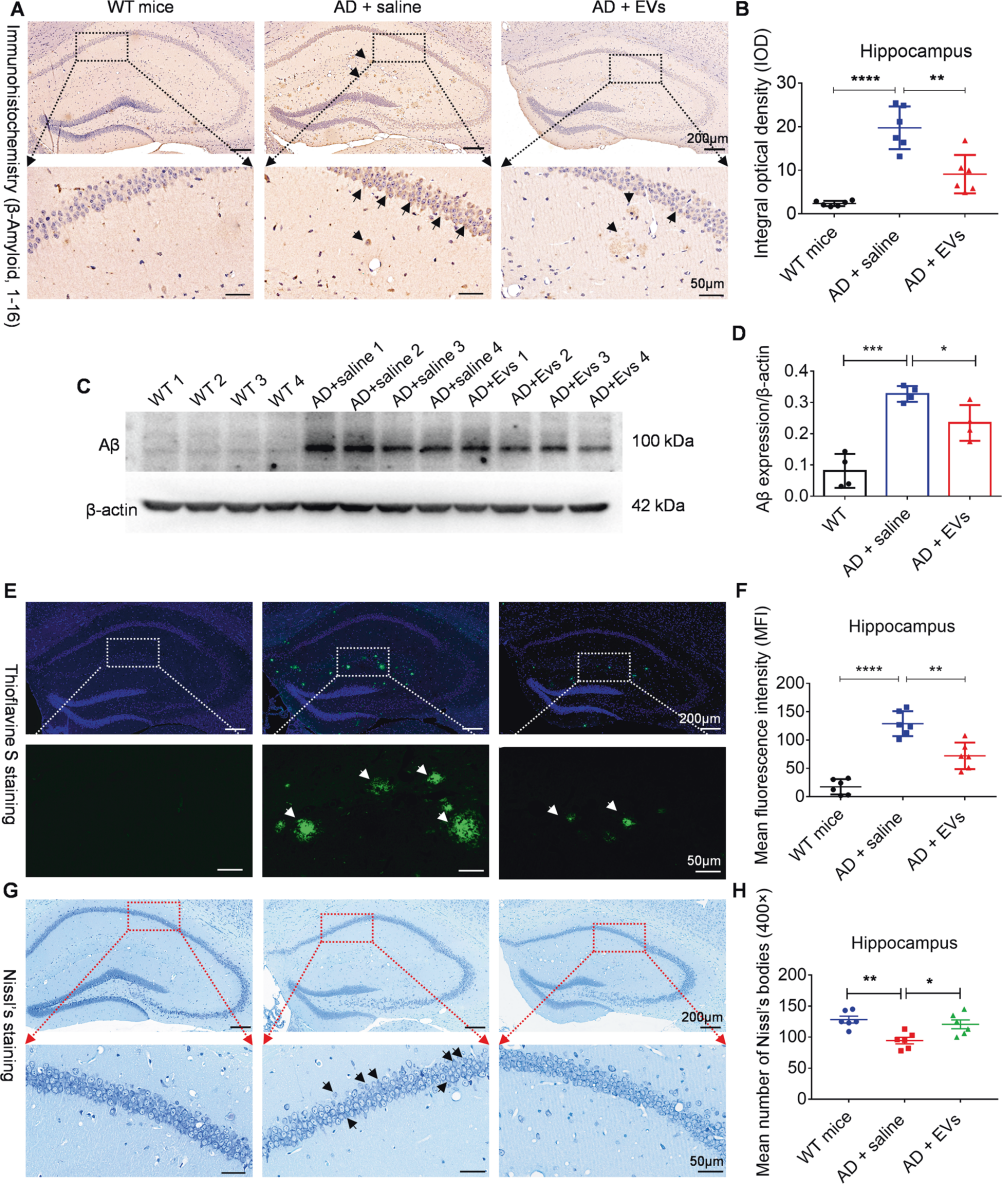
Tail vein injection of MSC-EVs reduces the hippocampal Aβ aggregation and neuronal loss in APP/PS1 mice. **A** After MSC-EVs injection for 1 month, A β amyloid 1, 16 (Aβ) immunohistochemistry images present the deposition of Aβ in the hippocampal regions of experimental mice (bar = 200 μm); areas in squares (up), at higher magnification (down) (bar = 50 μm), show the distinction of Aβ aggregation in hippocampus (black arrowheads) among three groups. **B** Histogram display the integral optical density (IOD) of Aβ aggregation in the experimental groups (*n* = 6). **C** Western blotting for the Aβ expression at 1 month in wild-type (WT) and APP / PS1 mice. **D** Statistic analysis show the relative expression of Aβ / β-actin in each group (*n* = 4 per group). **E** Thioflavine S staining present MSC-EVs-mediated changes in terms of Aβ aggregation in hippocampus (bar = 200 μm), and in the down panel, Aβ deposition (green, white arrowheads) is shown at higher magnification (bar = 50 μm). **F** Plaque quantification in hippocampus appears as mean fluorescence intensity (MFI) in each group (*n* = 6 per group). **G** Representative images show the Nissl’s staining in the experimental groups (bar = 200 μm), and the higher magnification of square areas display the Nissl’s body loss in hippocampus of each group (bar = 50 μm). **H** Histogram show the mean number of Nissl’s bodies (400×) in the experimental groups (*n* = 6 per group). Quantification data are expressed with Mean ± SEM. The data meet normal distribution and the variance is homogeneous. *P < 0.05, **P < 0.01, ***P < 0.001, ****P < 0.0001.

As it’s shown in Fig. 2A and C, our experiments revealed that, compared to the WT mice, Aβ deposition was increased in APP / PS1 mice as evidenced by the integral optical density (IOD) value (Fig. 2B, *P* < 0.0001) and protein expression (Fig. 2D, *P* < 0.001) of Aβ in the hippocampus. After MSC-EVs treatment, a reduction in Aβ expression was observed in AD + EVs mice than that observed in the AD + saline mice (Fig. 2B, *P* < 0.01; Fig. 2D, *P* < 0.05). Thioflavin staining was further used to probe the Aβ protein deposition in the experimental groups (Fig. 2E), and consistent with above results, the mean fluorescence intensity (MFI) in the hippocampus of the AD + EVs group was significantly lower than AD + saline group (Fig. 2F, *P* < 0.01). Moreover, Nissl’s staining (Fig. 2G) was employed to examine the neuronal loss in hippocampus, statistical analysis revealed that, compared to WT mice, the mean number of Nissl’s bodies presented a significant reduction in APP / PS1 mice (Fig. 2H, *P* < 0.01), while MSC-EVs treatments decreased the change in comparison to the saline group (Fig. 2H, *P* < 0.05). Together, these results imply that MSC-EVs reduce the Aβ aggregation and neuronal loss in hippocampus of the APP / PS1 mice.

### EVs treatment improves cognitive deficits in the APP / PS1 mice

**Fig. 3:**
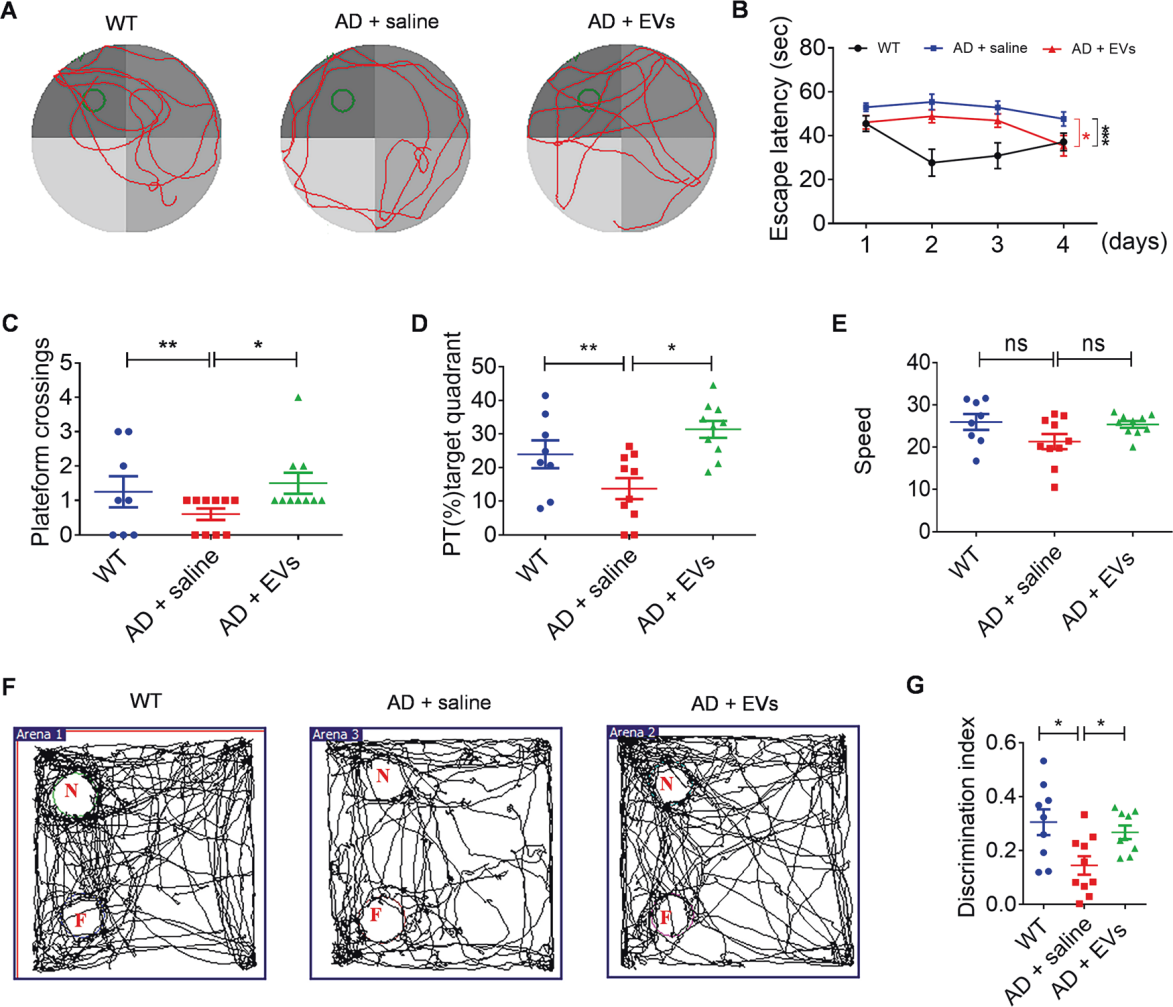
MSC-EVs treatment improves cognitive deficits in the APP/PS1 mice. **A** The trajectory of three groups mice in Morris Water Maze (MWM) test. **B** After MSC-EVs administration for 1 month, statistical analysis presents the spatial learning session and escape latency in the experimental groups. **C–E** In the probe test, histogram show the platform crossings (**C**), percentage of the total time (PT %)-target quadrant (**D**), and speed (mm / second) (**E**) in different experimental groups. **F** Motion trail of each group in novel object recognition test (NORT) (N novel object, F familiar object). **G** Comparison of the discrimination index of WT (*n* = 8), AD + saline (*n* = 10), and AD + EVs (*n* = 10) groups mice. The values represent Mean ± SEM. The data meet normal distribution and the variance is homogeneous. *P < 0.05, **P < 0.01, ^ns^*P* > 0.05.

After MSC-EVs injection for 1 month, MWM test (Fig. 3A) showed that, compared to the WT mice, the learning and memory impairments in the AD + saline group were characterized by a longer time of escape latency (Fig. 3B, *P* < 0.001) and a decrease of platform crossings (Fig. 3C, *P* < 0.01) and percent time (PT) in the target quadrant (Fig. 3D, *P* < 0.01). Whereas, an indication of behavioral improvements (including escape latency, platform crossings and PT in the target quadrant) were observed in the AD + EVs group (Fig. 3B-D, *P* < 0.05) compared to the AD + saline group. Additionally, there was no significant difference in the swim speed (Fig. 3E, *P* > 0.05) among the experimental groups, which excluded the influence of dyskinesia on the results. Furthermore, NORT examination (Fig. 3F) revealed that, compared to the WT mice, the AD + saline group displayed an inability for novel object discrimination (N, Fig. 3G, *P* < 0.05) as they spent a similar percentage of time exploring the familiar and novel objects. Notably, animals in AD + EVs group showed an increased discrimination index compared to the AD + saline group (N, Fig. 3G, *P* < 0.05). Together, these results indicate that MSC-EVs treatment improves the cognitive deficits observed in APP / PS1 mice.

### MSC-EVs treatment ameliorates calcium transients in Aβ-stimulated primary culture of hippocampal neurons

**Fig. 4:**
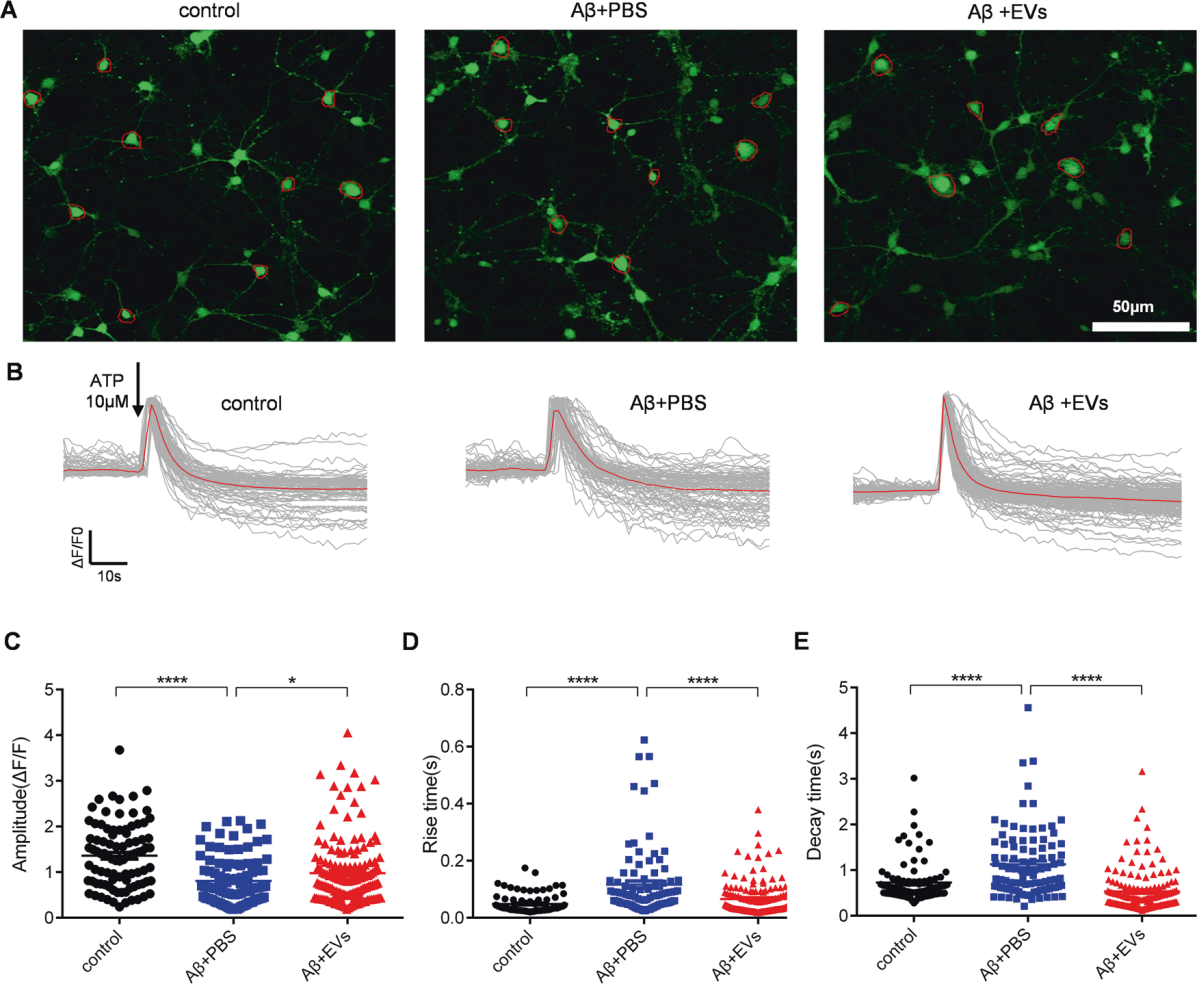
MSC-EVs therapy ameliorates calcium transients in Aβ-induced primary culture of hippocampal neurons. **A** Confocal microscope images of calcium signaling (green) in cultured primary neurons using Fluo-8 AM in control, Aβ + PBS, Aβ + EVs groups (bar = 50 μm). **B** Igor software assay shows the two phases (20 and 50 seconds, respectively) of the intracellular calcium transients in each group. **C–E** Statistical analysis data of the amplitude (ΔF/F) (**C**), rise time (**D**), and decay time (**E**) of the calcium signaling after adding ATP in each group (*n* > 70 cells per group). Data expressed as scatter plots with Mean ± SEM. The data meet normal distribution and the variance is homogeneous. *P < 0.05, ****P < 0.0001.

Calcium imbalance induced by amyloid Aβ drives the synaptic plasticity and neuronal loss observed in AD [19], we therefore assessed for calcium signaling alterations using an Aβ-induced primary culture of hippocampal neurons. As it’s shown in Fig. 4A, calcium imaging revealed differences in the fluorescence properties of hippocampal neurons among the experimental groups. After addition of ATP, the first phase calcium response consisted of a sharp peak in calcium signaling, followed by a second phase response of a slowly declining intracellular calcium concentration in each group (Fig. 4B). The statistical analysis revealed that, compared to the control group, Aβ stimulation resulted in a reduced amplitude of intracellular calcium transients (Fig. 4C, *P* < 0.0001) in the primary culture of hippocampal neurons after adding ATP, whereas MSC-EVs treatment significantly increased the calcium influx compared to the Aβ + PBS group (Fig. 4C, *P* < 0.05). We also found a slower change in the response rise time (Fig. 4D, *P* < 0.0001) as well as the decay time (Fig. 4E, *P* < 0.0001) in the Aβ + PBS group in compared to the control group. Remarkably, EV therapy (Aβ + EVs) reversed the rate of calcium transients observed in the Aβ + PBS group (Fig. 4D and E, *P* < 0.0001). Together, these data indicate that MSC-EVS treatment ameliorates the alterations in calcium transients in Aβ-stimulated primary hippocampal neurons.

### MSC-EVs administration repairs the neuronal morphology alterations in hippocampus

**Fig. 5:**
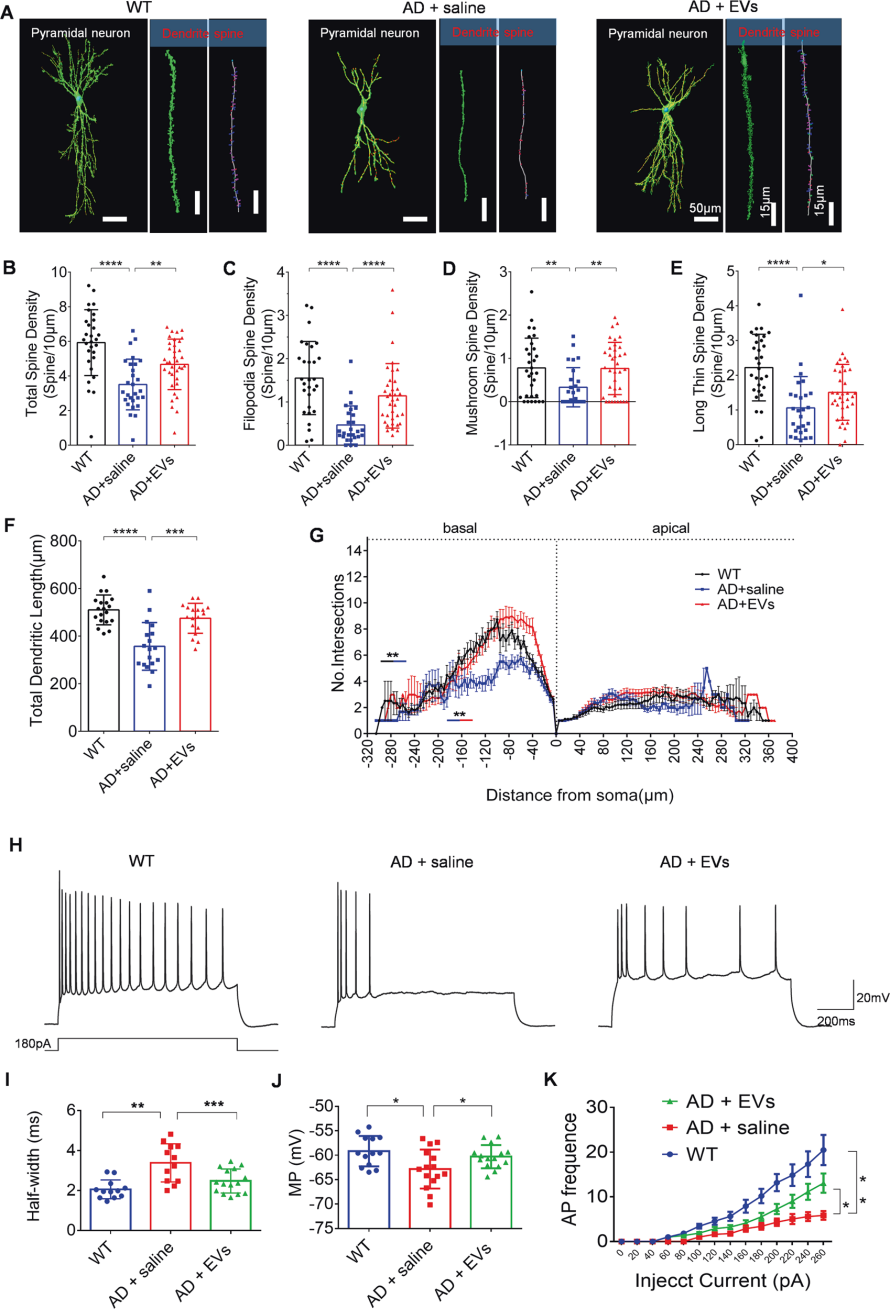
MSC-EVs administration restores the hippocampal neuronal morphology and function in mice. **A** Images show the 3D reconstruction of dendritic spine and dendritic phenotype for the hippocampal neurons in the WT, AD + saline, and AD + EVs group (left bar = 50 μm; right two bars = 15 μm). **B–E** Imaris analysis of spines along basal dendrites including total spine density (**B**), filopodia spine density (**C**), mushroom spine density (**D**), and long thin spine density (**E**) of three groups. **F**, **G** Statistical analysis of total dendritic length (**F**) and dendritic intersections (**G**) in the panel. **H** Trace images show the waveform of the pyramidal neurons in each group. **I–K** Histogram represent the statistical data of half-width (**I**), neuronal membrane potential (MP) (**J**), and action potential (AP) frequency (**K**) in each group (*n* = 15 per group). The values represent as Mean ± SEM. The data meet normal distribution and the variance is homogeneous. *P < 0.05, **P < 0.01, ***P < 0.001, ****P < 0.0001.

Synaptic morphology and plasticity are critical for neuronal function and are known to be associated with the memory impairment in AD [20]. Herein, we examined the dendritic processes and spines of hippocampal pyramidal neurons using Golgi staining at 1 months after EV treatment. The representative images displayed obvious morphological differences (Fig. S3A) including dendritic complexity and three-dimensional restoration of spines (Fig. 5A), in addition to dendritic phenotype (Fig. S3B) among the WT, AD + saline, and AD + EVs groups. 3D reconstruction of dendrites showed a reduction of total spine density (Fig. 5B, *P* < 0.0001), and filopodia / mushroom / long thin spine density (Fig. 5C-E, *P* < 0.01) in hippocampal pyramidal neurons in APP / PS1 mice compared to WT mice. Additionally, after neuronal phenotype Sholl analysis, we found that APP / PS1 mice had lower total dendritic length (Fig. 5F, *P* < 0.0001) and lower dendritic intersections of basal dendrites (−40–180 μm) (Fig. 5G, *P* < 0.01) than WT mice. Remarkably, these morphological changes in pyramidal neurons were reversed in the AD + EVs group compared to the AD + saline group (Fig. 5B-E and F-G, *P* < 0.05). Together, these results suggest that the morphological alterations observed in hippocampal neurons of APP / PS1 mice are restored by MSC-EVs therapy.

### EV therapy restores hippocampal neuronal excitability and mitochondrial changes in the AD model

**Fig. 6:**
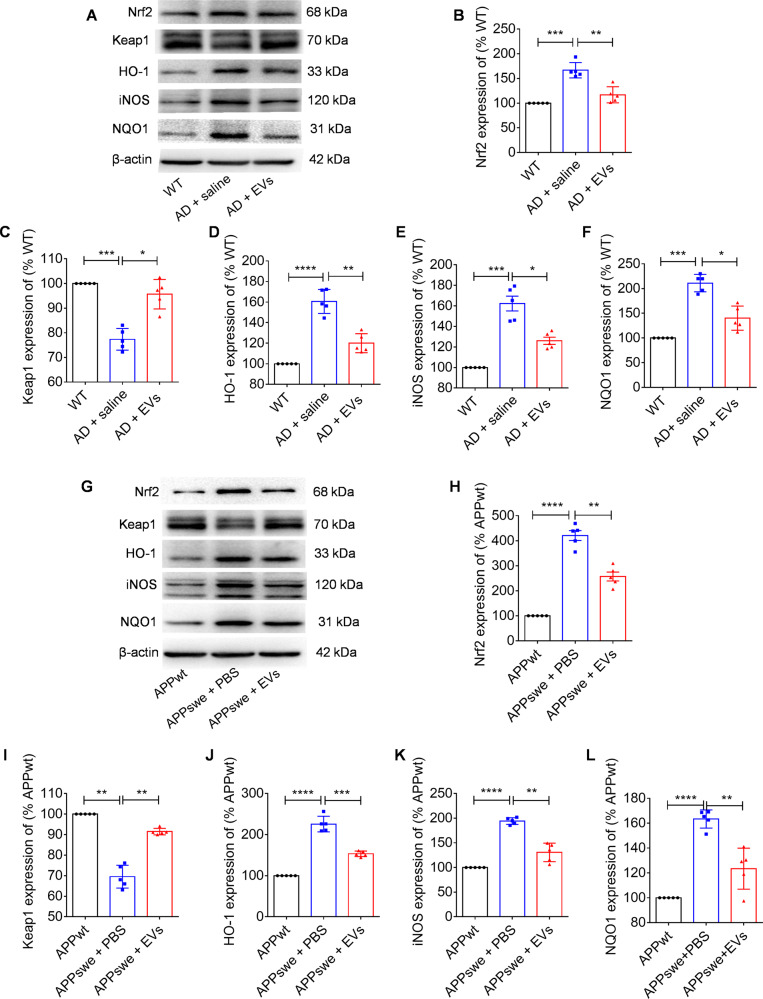
Oxidative defense system is associated with the EVs therapy in APP/PS1 mice. **A** Western blots of nuclear factor E2-related factor 2 (Nrf2) signaling and oxidative marker inducible nitric-oxide synthase (iNOS) in the animal models. **B–F** Statistical analysis of the relative expression of Nrf2 (**B**), Keap1 (**C**), HO-1 (**D**), iNOS (**E**), and NQO1 (**F**) in the WT, AD + saline, and AD + EVs groups mice (*n* = 5 per group). **G** Western blots of Nrf2 signaling and iNOS in APPwt, APPswe + PBS, and APPswe + EVs groups. **H–L** Statistical analysis of the relative expression of Nrf2 (**H**), Keap1 (**I**), HO-1 (**J**), iNOS (**K**), and NQO1 (**L**) in cell models. The values represent Mean ± SEM. The data meet normal distribution and the variance is homogeneous. *P < 0.05, **P < 0.01, ***P < 0.001, ****P < 0.0001.

Hippocampal CA1 pyramidal cells implicate the progression of neuronal dysfunction and cognitive deficits [21], we thus used whole-cell patch-clamp recording to detect the neuronal excitability in the experimental groups (Fig. S4). After analyzing the waveform of hippocampal CA1 pyramidal neurons (Fig. 5H), we found an increase in half-width (Fig. 5I, *P* < 0.05) and a decrease in membrane potential (Fig. 5J, *P* < 0.05) and frequency (Fig. 5K, *P* < 0.01) of action potentials in the AD + saline group compared to the WT mice, whereas EVs treatment significantly reversed (Fig. 6I-K, *P* < 0.05) these potential changes, suggesting that MSC-EVs treatment restores the excitability of hippocampal CA1 pyramidal neurons in APP / PS1 mice. Importantly, mitochondria are essential for the maintenance of energy in hippocampal neurons [22]. We therefore examined the ultrastructure (Fig. S5A) and injury markers (Fig. S5B) of mitochondria in each group. As shown in TEM images (Fig. S5A), the mitochondria in hippocampal neurons of AD mice exhibited obvious swelling and vacuolation (red arrow) compared with the WT mice (white arrow), while a trend toward normal mitochondrial structure was observed in the AD + EVs group (blue arrow). Moreover, compared to the WT mice, the altered mitochondrial fission / fusion was typified by an increase in COX IV (Fig. S5C, *P* < 0.001), Tom20 (Fig. S5D, *P* < 0.001), and FIS1 (Fig. S5E, *P* < 0.001) expression in the hippocampal tissue of AD mice. Notably, after MSC-EVs treatment, these protein markers were found to be reduced in comparison to the AD + saline group (Fig. 6C-E, *P* < 0.01). Combined, these results suggest that EV therapy ameliorates the mitochondrial changes observed in APP / PS1 mice.

### Nrf2 defense system is associated with the outcome of EV therapy in APP / PS1 mice

Oxidative stress is believed to be a cause of neuronal degeneration in Alzheimer’s disease, while Nrf2 play a pivotal role in the mediation of oxidative stress [16]. We first investigated whether Nrf2 signaling participates in the action of MSC-EVs in APP / PS1 mice. As shown in Fig. 6A, compared with age-matched control mice, the AD mice (AD + saline) displayed a remarkably higher level of Nrf2 (Fig. 6B, *P* < 0.001), HO-1 (Fig. 6D, *P* < 0.001), NQO1 (Fig. 6E, *P* < 0.001), iNOS (Fig. 6F, *P* < 0.001), and a lower level of Keap1 protein expression (Fig. 6C, *P* < 0.001), while EVs treatment reversed the expression of these proteins (Fig. 6B-F, *P* < 0.05) that observed in the hippocampus of AD model. To further confirm the participation of this signaling in the action of MSC-EVs on the hippocampal neurons, AD cell models including the SHSY5Y (APPwt) and SHSY5Y (APPswe) cell lines were employed in an in vitro study. As can be seen in Fig. 6G, Nrf2 signaling (Nrf2, Keap1, HO-1, NQO1) and iNOS exhibited variations similar to those observed in the in vivo studies between the APPwt and APPswe + PBS group (Fig. 6H-L, *P* < 0.01). Remarkably, MSC-EVs administration significantly reduced the expression of Nrf2 (Fig. 6H, *P* < 0.01), HO-1 (Fig. 6J, *P* < 0.001), iNOS (Fig. 6K, *P* < 0.01), NQO1 (Fig. 6L, *P* < 0.01), and increased the expression of Keap1 in the APPswe + EVs group (Fig. 6I, *P* < 0.01) compared to the APPswe + PBS group. Together, above results indicate that Nrf2 defense system participates in the action of MSC-EVs on the neuronal deficits in APP / PS1 mice.

## Discussion

Our results show that MSC-EVs treatment inhibits the deposition of Aβ protein and neuronal loss observed in the hippocampus of AD mice as well as ameliorates the deficits in neuronal structure and function typified by the calcium transients, morphology alterations, mitochondrial changes, excitability abnormalities, and associated cognitive repairments observed in Aβ-stimulated primary culture or APP / PS1 mice. Additionally, the present data demonstrated that the Nrf2 signaling pathway participates in the actions of MSC-EVs on neuronal damage in AD using cell and animal models. Together, these results suggest that MSC-EVs can represent a functional nanotherapeutic agent for the treatment of AD.

Since the progressive cognitive impairments are the typical symptoms in AD [1], we firstly performed MWM and NORT tests to detect the behavioral outcome in the experimental groups. Our results showed that EVs treatment improved their behavioral performances including learning, memory, and recognition compared to the saline group, suggesting that the positive therapeutic effects of MSC-EVs on cognitive deficits in APP / PS1 mice. Notably, Aβ aggregation is a typical pathological feature in AD patients and animal models, while the hippocampi are responsible for the memory storage, recognition and other brain functions [23]. To clarify the administered MSC-EVs produced pathological improvements on APP / PS1 mice, we performed a systematic investigation of deposited amyloid plaques in the hippocampus of each group. Significantly, our histological and biological study showed that EV therapy reduces the expression of Aβ related indications, implying the positive effects of MSC-EVs on Aβ aggregation in the hippocampus of APP / PS1 mice. Moreover, Aβ is believed to be a crucial and primary factor in triggering progressive neuronal loss in AD [24], the Nissl’s staining revealed that the hippocampal neuronal loss can be reversed by MSC-EVs injection in the present study. Together, the above results suggest that MSC-EVs show markable therapeutic effects on behavioral deficits and pathological changes in APP / PS1 mice.

Among the multiple features of AD initiation and progression, neuronal deficits are believed to be the ultimate cause of cognitive decline, and the hippocampi are susceptible to Aβ aggregation in AD [23, 24]. Thus, to probe the underlying mechanism of MSC-EVs therapy in APP / PS1 mice, we further detect the neuronal structure and functional reconstruction in the experimental groups. Synaptic transmission provides the physiological, cellular, and molecular mechanisms for cognitive function, while the synaptic deficits are reflected by dendritic density and complexity in neuronal cells [25]. Thus, extensive loss of synapses and dendritic spines can contribute the neuronal dysfunction and cognitive impairments in AD [26]. In the present study, after EV therapy, the morphological changes in spine density and dendritic intersections implied that the structural impairments in hippocampal CA1 pyramidal neurons can be restored by MSC-EVs in APP / PS1 mice. Furthermore, calcium is a ubiquitous intracellular messenger and acts as a key regulator for cell homeostasis [27]. In AD, the neuronal dysfunction can be reflected by the calcium signaling alterations, while calcium dysregulation also results in the neurodegeneration and memory defects [13, 19, 28]. We thus used calcium imaging to investigate the iron transients in the primary culture and observed increased amplitude and a faster change in the response after MSC-EVs addition, suggesting that MSC-EVs ameliorated Aβ-stimulated calcium transients in the primary culture of hippocampal neurons, which may facilitate to restore the cognitive deficits in AD. Markedly, abnormal excitability is a well-known alteration that occurs in AD [29, 30]. Our whole-cell patch-clamp experiment is consistent with this as the electrophysiological activity of hippocampal neurons was altered in APP / PS1 mice, evidenced by a decreased frequency of APs and membrane potentials (MPs). Whereas, MSC-EVs administration reversed this alteration in neuronal excitability suggesting that EVs treatment restores the electrophysiological function of hippocampal neurons compared to the AD + saline group. Also, mitochondrial dysfunction is involved in the pathogenesis of most nervous system diseases including AD [31]. Here, we used TEM to detect the mitochondrial ultrastructure and protein assay to examine mitochondrial fission/fusion in the experimental groups. Compared to the saline group, the ameliorated mitochondrial ultrastructure and decreased COX IV, Tom20, and FIS1 expression observed in AD + EVs group, indicated that EVs administration ameliorates the mitochondrial changes in APP / PS1 mice. Together, these results suggest that the abnormalities (especially for the structure and function) in hippocampal neurons observed in cell or animal models can be restored by MSC-EVs, which provides new evidence for the nanotherapeutic action of EVs in AD mice.

Remarkably, hippocampal neurons are highly susceptible to oxidative stress in AD patients and animal models, and the redox imbalance is considered to be a prominent factor of neuronal damage in the brain [14, 16]. Taking this into consideration, examination of the oxidative defense system may reveal the mechanism(s) underlying MSC-EVs therapeutic action on hippocampal neurons in AD. Furthermore, our previous study indicated that MSC-EVs show antioxidant activity as they contained enriched functional agents, such as nucleic acids, proteins, and enzymes that regulate redox reactions [32]. Antioxidants also have great potential in the treatment of neurodegenerative diseases and have been shown to improve learning and memory deficits in AD [33]. Among the multiple molecular pathways implicated in the oxidative defense system, Nrf2 is an essential element for the regulation of oxidative responses in neuronal degeneration [16]. Under physiological conditions, Nrf2 interacts with the cytoplasmic protein Keap1, whereas it is isolated from Keap1 and translocated into the nucleus under stress conditions [34]. Numerous studies have indicated that Nrf2 can activate transcription of its target genes HO-1 and NQO1, which then exerts a cytoprotective effect against neuronal damage [35]. In the present study, compared with AD + saline group, MSC-EVs treatment resulted in a decrease of Nrf2, HO-1, NQO1, iNOS (indicative of oxidative damage), and an increase of Keap1 expression in hippocampal tissue. Also, these changes were observed in the AD cell models (SHSY5Y, APPswe) after EVs administration. Even though several papers reported that an apparent decrease can be shown in Nrf2 activity in APP/PS1 mice, many data supported that Nrf2 presented upregulation in the hippocampal cells / tissues of AD brain and animal models [36, 37]. Exactly, Nrf2 activity varies among animal models, regions, and timepoints. Here, we reported that the upregulated Nrf2 expression in APPswe cell line and the hippocampus of 10-month-old APP/PS1 mice could be reduced by EV therapy. Notably, prolonged and strong activation of NRF2 can causes tissue damage, cancer progression, or chemoresistance [38], an adaptive activation of NRF2 is more feasible to protect cells from stress stimulation [39]. Combined, these results suggest that the therapeutic effects of MSC-EVs on hippocampal neuronal dysfunction might be regulated by Nrf2 signaling pathway.

In summary, the present study shows that MSC-EVs treatment ameliorates the hippocampal neuronal deficits observed in cell model or APP / PS1 transgenic mice, and that the therapeutic mechanism is associated with the Nrf2 defense system in vitro and in vivo, suggesting that MSC-EVs can serve as functional nanotherapeutic agents in the treatment of AD.

## Materials and methods

The supplement information document (SI Materials and Methods) (Figs. S1 and 2; Table S1) comprises description of all materials and methods [32, 40–45] employed in this study.

## Supplementary information

Supplemental information

Dataset 1

## Data Availability

All data, models, and code generated or used during the study appear in the submitted article.
