# Supplementary material for: Tail-vein injection of MSC-derived small extracellular vesicles facilitates the restoration of hippocampal neuronal morphology and function in APP / PS1 mice
Source: Cell Death Discov. 2021 Sep 4;7:230. doi: 10.1038/s41420-021-00620-y (PMC8418600; doi:10.1038/s41420-021-00620-y)

**Supplementary information**

**SI Materials and Methods**

***Animals***

APPswe / PS1dE9 (APP / PS1) transgenic mice (male, SPF grade) and age-matched wild type (WT) littermates were purchased from Beijing HFK Bioscience Co., LTD. The APP / PS1 mice over-express the delta exon 9 variant of presenilin 1 (PS1) and the Swedish mutation of β-amyloid precursor (APP) (Figure S1). For the primary neuron culture, pregnant (embryo at 15~18-day) C57B / 6 mice were obtained from the Experimental Animal Center of the Fourth Military Medical University. The experimental protocol was performed in accordance with the guidelines for the International Council for Laboratory Animal Science (ICLAS) and approved by the Ethics Review Board of Fourth Military Medical University. Animals were housed in a controlled environment under conventional conditions with a 12 h light / dark cycle and food and water provided ad libitum. The animals in each group were selected at random and conducted in a single blind method. In order to ensure the repeatability of the experiment and control the number of the required animals, 8~10 mice in each group were used for behavioral test and 5 ~ 6 mice in each group were selected for immune-staining, patch clamp or Western blotting. A total of 70 (including 45 APP/PS1) mice were used in the present study.

***Cell preparation***

For preparation of MSCs, a signed consent form was obtained from the donors with full-term puerpera in good health, and human umbilical cord tissues were harvested from fetuses with natural labor. The procedures were approved by the Guideline of the Ethical Committee of the Xi’an Central Hospital, Xi’an Jiaotong University (No. 20180715-1), in accordance with the principles stated in the Declaration of Helsinki. MSCs was obtained *via* enzymatic digestion of Wharton’s jelly within the human umbilical cord and expanded using standard medium, α-minimum essential medium (α-MEM; Gibco, New York, USA) supplemented with 10% fetal bovine serum (FBS; Gibco, New York, USA), 100 U/mL penicillin and 100 μg/ mL streptomycin (Gibco, New York, USA) as our previous reports [18].

The primary culture of hippocampal neurons was collected from hippocampal tissues. Briefly, hippocampal tissue was stripped from 16–18-day old embryos of mice in a sterile environment and digested at 37 ℃ with 0.125% trypsin for 10 min and then stopped by Dulbecco’s modified Eagle’s medium (DMEM) containing 10 % FBS. The number and concentration of viable cells were determined by trypan blue exclusion using an automated cell counter (JSY-SC-031, BodBoge, China). Whereafter, the cells were plated in neuronal growth and maintenance medium containing neurobasal medium (Gibco, New York, USA), 2% B27 (Gibco, New York, USA), 100 U/ml penicillin-streptomycin, and 2 mM L-glutamine (Gibco, New York, USA).

Two SH-SY5Y cell lines (APPwt and APPswe), expressing wild type Aβ precursor protein (APP) and APP with the Swedish mutation, respectively, were obtained from iCell Bioscience Inc, Shanghai and authenticated by short tandem repeat (STR) profiling (Table S1). SH-SY5Y APPwt cells generate minor quantities of Aβ peptides while the SH-SY5Y APPswe cells produce high concentrations of Aβ [19]. The cells were grown in DMEM supplemented with 10% FBS, 1% MEM Non-Essential Amino Acids, 1% penicillin / streptomycin in 5% CO_2_ in air at 37 °C in a humidified incubator.

***MSC-EVs isolation, characterization and labeling***

For preparation of MSC-EVs, the procedures were according to our previously described [18, 20]. In brief, the 5th passage of MSCs were cultured with EV-depleted FBS for 48 hours (h), a series of ultracentrifugation was employed to concentrate the EVs in the supernatants, and the pellet was washed with ice-cold PBS, resuspended in PBS and stored at -80℃. EVs quantification was performed by determination of the protein contents using a bicinchoninic acid assay (BCA; Beyotime, P0010, Beijing, China). While the size distribution and morphology of the EVs were detected by nanoparticle tracking analysis (NTA; Nanosight LM10, Malvern, Worchestershire, UK) and transmission electron microscopy (TEM; JEM-1400, JEOL Ltd., Japan), respectively. Also, Western blotting was used to assay the representative markers of EVs including CD63 (1:200) (sc-5275, Santa Cruz, USA), CD9 (1:1000) (ab92726, Abcam, California, USA), TSG101 (1:5000) (ab30871, Abcam, California, USA) and calnexin (1:1000) (AC018, Beyotime, Beijing, China). In addition, C5 Maleimide-Alexa 594 (CM-A954) (Invitrogen, A10256, California, USA) was used to label MSC-EVs as previously described [21].

***MSC-EVs administration and tracking***

For *in vitro* experiments, 10 μg/ml MSC-EVs (Figure S2A) or an equivalent volume of PBS were added to the culture medium of the primary culture of hippocampal neurons and SHSY5Y APPswe cells (APPswe + EVs or APPswe + PBS group), and then incubated for 12 h to ensure incorporation of the EVs. The hippocampal neurons were subsequently exposed to 10 μM Aβ 1-42 (AnaSpec, San Jose, CA, USA) and PBS for 24 h as Aβ + EVs and Aβ + PBS group based on previous study [22]. For *in vivo* studies, nine-month-old APP / PS1 mice were randomly injected with 50 μg MSC-EVs (diluted in 150 μl saline) (Figure S2B and C) or 150 µL of saline *via* tail vein as AD + EVs or AD + saline group, respectively. Age-matched WT mice received an equivalent volume of saline as the WT group. Also, the animal models (n = 3) were administered by CM-A594-labelled MSC-EVs to track the vesicles in the hippocampal neurons *in vivo*.

***Behavioral test***

After AD model received MSC-EVs for 1 month, the Morris water maze (MWM) was used to test spatial memory and cognitive function of WT (n = 8), AD + saline (n = 10) and AD + EVs group (n = 10). The tests were conducted in a water maze drum filled with milky white water dyed by edible pigment at 20 °C ± 1 °C. Spatial learning sessions were performed over four consecutive days with four trials per day. A small escape platform was submerged 1–2 cm under water surface. The mice were gently placed into the water, facing the wall, at the corresponding entry point in a quasi-random fashion to prevent strategy learning, free to find the platform. Each trial lasted until the animal found the platform or for a maximum of 60 seconds. After every trial, the mice were placed in a clean, warm environment and given food and water to replenish their energy. A 60 seconds probe test was conducted 24 h from the last learning session, during which, the hidden platform that was present for the first four days was removed. Mice were allowed to swim freely in the water. A tracking system (Morris2.8.1, China) was used that automatically recorded and analyzed the latencies to reach the platform, the activity time, the percentage of the total time (%, PT) in the target quadrant, and the frequency of mice crossing the platform.

Additionally, the novel object recognition test (NORT) was conducted to measure recognition memory. Mice were individually placed in a square open field apparatus with a side length of 44 cm, free to explore for 5 min in the habituation session, and then returned to their home cage. After each experiment, the open filed was thoroughly cleaned with 75% ethanol to minimize olfactory cues before the next mouse entered the open field. In the familiarization phase performed 24 h after the first session, two identical objects were placed in two opposite areas of the apparatus 10 cm away from the wall, and the mice were again placed in the field with their head positioned opposite the objects to explore for 10 min. Twenty-four h later, the test session was conducted. One of the objects was replaced with a novel one and mice were placed in the arena containing one familiar and one new object to explore for another 10 min. Their behavior and exploring time were recorded by a video tracking system for analysis. A discrimination index was calculated as follows: Discrimination index = (time on novel – time on familiar) / (time on novel + time on familiar). All operations were conducted between 8 a.m. and 5 p.m.

***Histological study***

After above behavioral tests, the animals (n = 5 per group) were sacrificed with isoflurane and perfused intracardially with cold 4 % paraformaldehyde solution. After that, the brain sections through the hippocampus (bregma from -2.64 mm to -3.48 mm) were selected and processed for histological study as previously described [12, 20]. For immunostaining, the primary antibodies, including purified anti-β-Amyloid, 1-16 antibody (1:500) (Biolegend, SIG-39320, San Diego, CA, USA), and mouse anti-NeuN (1:100) (Abcam, Ab104224, California, USA) were diluted in blocking solution and incubated with sections overnight at 4 °C. After incubation, the following secondaries biotinylated anti-mouse [heavy- and light-chains (H+L); BA-2000; Vector Lab] or A488 anti-mouse IgG (1:500) (Thermo Fisher Scientific, A-32790, New York, USA) were used. DAPI (Sigma-Aldrich, 32670, California, USA) was used for cell nuclei counterstaining, and the peroxidase reaction was developed using a diaminobenzidine substrate. To detect Aβ plaques in the hippocampus, 0.3% thioflavin S solution was diluted in 50% alcohol (volume / volume) and filtered, and then incubated for 8 min at room temperature. The prepared sections were washed in 80% alcohol for 10 seconds and stained with 0.3% thioflavin S solution twice for 10 seconds each. Subsequently, the sections were rinsed with distilled water and cover-slipped with neutral balsam. To verify the neuronal loss in the hippocampus, Nissl’s staining was performed as our previously described [23]. Sectioned slices were treated with a solution of 0.5% toluidine blue and 1% sodium borate (pH 9.3) for 25 min. The Nissl’s bodies were calculated at least 10 counting frames (400× visual field) of each slice at random. All immuno-stained sections were photographed under a confocal microscope (Olympus, FV10-ASW, Japan) or a light microscope (Leica, DMi8, Germany), and analyzed using ImageJ Pro Plus V 6.0 (Bethesda, Maryland, USA).

***Western blotting***

Cell samples from the APPwt, APPswe + PBS, APPswe + EVs groups (n = 5 per group) and hippocampal tissues at 1 month after MSC-EVs administration from animals belonging to WT, AD + saline and AD + EVs groups (n = 5 per group) were dissected for Western blotting. Samples were lysed in Radio-Immunoprecipitation Assay Lysis Buffer (Beyotime, P0013B, Shanghai, China). Total protein samples were quantified using the BCA protein assay (Beyotime, P0012, Shanghai, China), and then normalized protein samples were separated *via* sodium dodecyl sulfate-polyacrylamide gel, and transferred to polyvinylidene fluoride membranes (Millipore, MA, USA). Membranes were blocked in tris-buffered saline containing 0.1 % Tween 20 (TBST) and 5 % nonfat milk at room temperature for 2 h. Membranes were incubated with primary antibodies including Aβ (1:2000), TOM20 (1:5000) (Proteintech, 11802-1-AP, USA), mitochondrial fission protein 1 (FIS1, 1:1000) (Proteintech, 10956-1-AP, USA), cytochrome c oxidase Ⅳ (COX Ⅳ, 1:5000) (Proteintech, 11242-1-AP, USA), Nrf2 (1:1000) (Abmart, T55136S, Shanghai, China), hemeoxygenase-1 (HO-1, 1:1000) (Proteintech, 10701-1-AP, Illinois, USA), kelch-like ECH-associated protein 1 (Keap1, 1:1000) (Proteintech, 10503-2-AP, Illinois, USA), NQO1 (1:1000) (Abmart, T56710M, Shanghai, China), inducible nitric-oxide synthase (iNOS, 1:1000) (Proteintech, 18985-1-AP, Illinois, USA), or β-actin (1:10000) (Abclonal, AC026, Wuhan, China) overnight at 4 °C, followed by three TBST washes. Membranes were then incubated with HRP-conjugated anti-mouse secondary antibodies (CW0102S, CWBIO, China), or HRP-conjugated anti-rabbit secondary antibodies (EK020, Zhuangzhi Biotech, China) for 2 h. Images were taken using a Bio-Rad imaging system (Bio-Rad, Hercules, California, USA) and analyzed by the Quantity One software package (West Berkeley, California, USA).

***Calcium imaging***

To investigate the neuroprotection of MSC-EVs on hippocampal neurons in response to Aβ stimulation, intracellular calcium oscillations in each group (control, Aβ + PBS, and Aβ + EVs group; n = 5 per group) were detected using the calcium indicator Fluo-8 AM (Abcam, AB142773, California, USA) as our previously described [20]. To load the dye into the hippocampal neurons, the primary cultures were rinsed in phenol red-free DMEM containing 4 μM Fluo-8 AM and 0.08% Pluronic F127 (Life Technologies, CA, USA) for 20 min at 37 °C. Subsequently, the cells were washed three times with Hank’s balanced salt solution and the samples were stored in artificial cerebrospinal fluid (ACSF) containing 124 mM NaCl, 25 mM NaHCO3, 2.5 mM KCl, 1 mM KH2PO4, 2 mM CaCl2, 2 mM MgSO4, and 10 mM glucose. Resting state calcium levels were recorded for 20 seconds, and then 10 mM adenosine monophosphate (ATP) was added into the ACSF to stimulate a calcium influx. The fluorescence images were captured every second for 70 seconds at the wavelength of 488 nm using a confocal microscope (Olympus, FV3000, Japan). The image analysis software Cellcens (Olympus, Japan) was used to measure calcium influx and resting calcium levels of the individual hippocampal neurons. More than 80 cells from at least three independent experiments were analyzed for each group using Igor Pro software (WaveMetrics in Oregon, USA).

***Golgi-Cox Staining***

Golgi-Cox staining was used to observe the morphology of neuronal dendrites as previous report [24]. The animals (n = 5 per group) were anesthetized and perfused with a 0.9 % sterile saline solution. The brains were removed and stained with Golgi-Cox solution (consisting 5 % potassium chromate, 5 % potassium dichromate, and 5 % mercuric chloride) and stored at room temperature in the dark for 2~3 days. After that, the solution was replaced with fresh Golgi-Cox solution for another 2 weeks. Then the brains were kept in a 25~30% sucrose solution for 2 days in order to reduce the tissue fragility during the sectioning process. After collection of 100~200-μm-thick coronal slices using a vibratome (Leica, VT1000s, Germany), the brain sections were washed in deionized water for 1 min, placed in 50 % NH_4_OH and subsequently in fixing solution (Kodak; Rochester, NY, USA) for 30 min. The sections were subsequently immersed in 5 % sodium thiosulfate for 10 min. After being rinsed with distilled water, the slices were dehydrated using increasing concentrations of ethanol. 5 slices were randomly selected in each mouse for the observation and analysis. All sections were photographed under the bright field of a confocal microscope (FV1000, Olympus, Japan) with an excitation wavelength of 405 nm. Images were taken by z-stack scanning, and then set the visible light to green.

***Whole-cell patch-clamp recording***

Brain tissue of experimental animals (n = 6 per group) was cut into 300 μm hippocampal coronal slices. An action potential was induced by positive current injection, which brought the membrane potential to -50 mV– -55 mV. The slices were immersed in oxygenated (95 % O_2_ / 5 % CO_2_) ACSF at room temperature until use, and then placed in a recording chamber and perfused with extracellular solution containing 124 mM NaCl, 24 mM NaHCO_3_, 2.5 mM KCl, 1.2 mM NaH_2_PO_4_, 2 mM MgSO_4_, 2 mM CaCl_2_, 5 mM HEPES, and 12.5 mM glucose. Patch pipettes, pulled with a tip resistance of 6-8 MΩ, were ﬁlled with internal solution (130 mM K+-gluconate, 0.3 mM EGTA, 4 mM KCl, 10 mM HEPES, 4 mM ATPMg, and 0.3 mM Na2-GTP, pH 7.4). Recording data were ﬁltered at 2 kHz, sampled at 10 kHz with a Digidata 1440 and Clampex 10.6 (Molecular Devices, USA) and acquired and analyzed using pClamp10.6 software (Molecular Devices, USA).

***Statistical analysis***

We were blinded to the group allocation during the experiment and assessing the outcome, all the data are presented as Mean ± SEM. Multiple comparisons were analyzed using one-way analyses of variance (ANOVA) or unpaired, two-tailed Student’s t-test. A repeated measures ANOVA was carried out to analyze the differences in escape latency among groups after Bonferroni posttest using SPSS 24.0.0 and GraphPad Prism 6 software (GraphPad Prism, USA). *P* values of less than 0.05 were considered statistically significant.

**Figure S1. Identification of APP / PS1 transgenic mice**


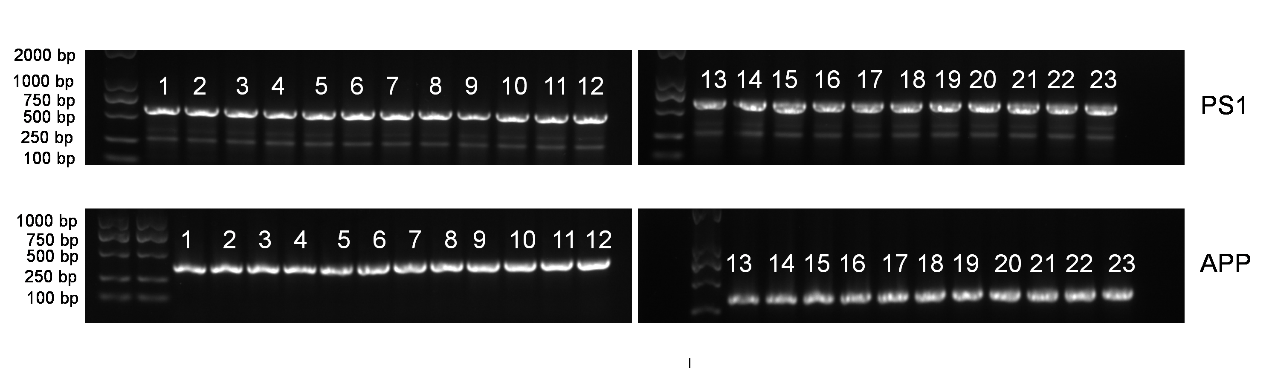


The RNA was isolated from the tail tissue of purchased transgenic mice and processed for PCR examination, the results show the mice (n = 23), selected at random, over-express the delta exon 9 variant of presenilin 1 (PS1, ≈ 600 bp) and the Swedish mutation of β-amyloid precursor (APP, ≈ 300 bp).

**Figure S2. Dose response of MSC-EVs *in vitro* and *in vivo***


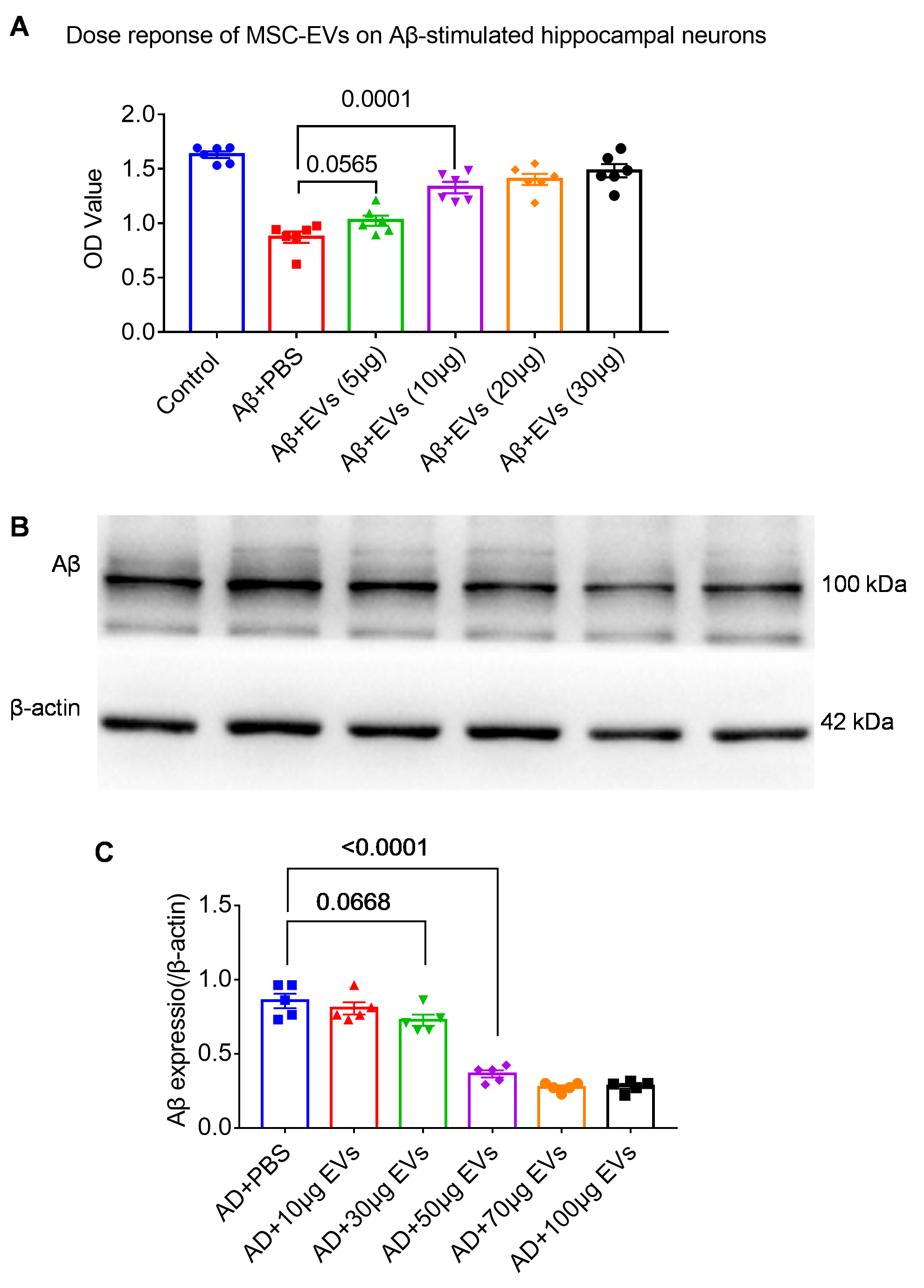


(A) CCK-8 assay shows the cell viability in Aβ-stimulated primary culture of hippocampal neurons administered by different dose of MSC-EVs (n = 6 per group). Note a significant increase of optical density (OD) value in 10 μg / ml MSC-EVs treated group in comparison to Aβ + PBS group. (B) After different dose of MSC-EVs injection for 1-month, Western blot of Aβ expression in APP / PS1 mice. (C) Histogram shows the dose response of MSC-EVs in AD mice model, the minimal effective concentration (50 μg MSC-EVs per mouse) was employed in this study. Statistical difference between groups is displayed in histograms.

**Figure S3. Golgi-Cox staining and neurite imaging**


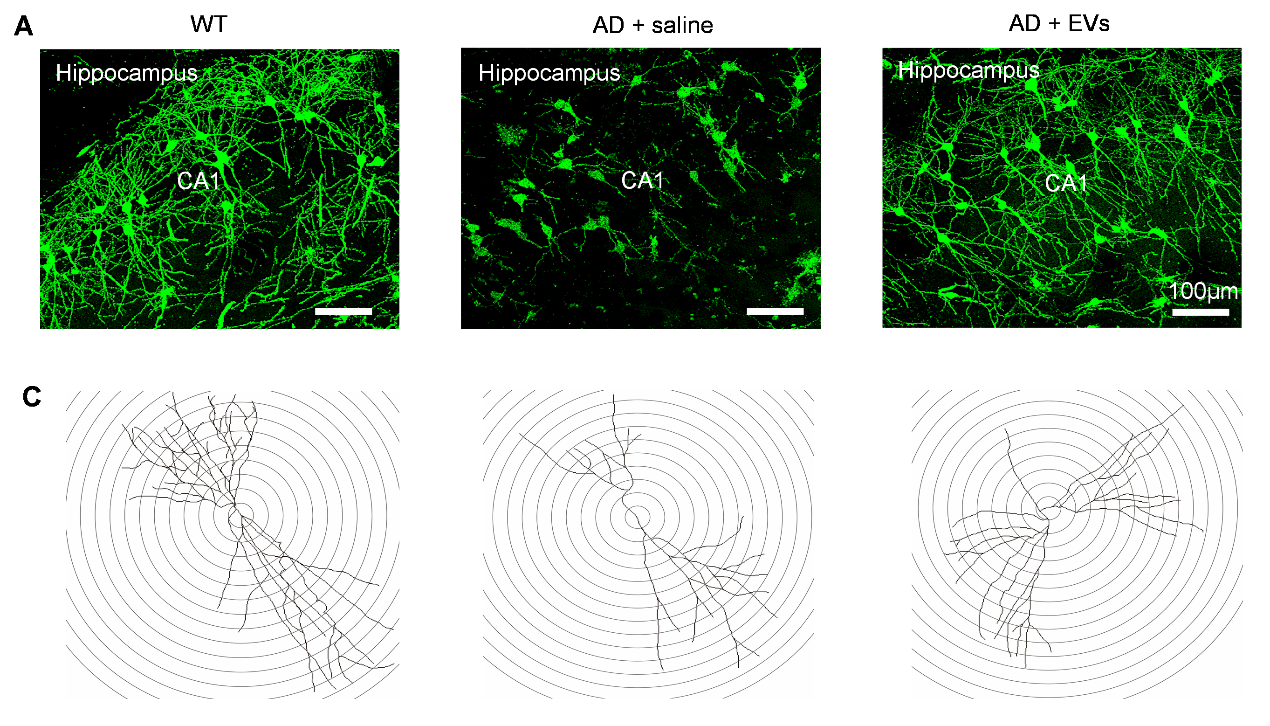


(A) Representative Golgi-Cox staining images of the neuronal morphology in the CA1 region of each group (bar = 100 μm). (B) Sholl analysis for neurite images of pyramidal neurons in naive, AD + saline and AD + EVs group.

**Figure S4. Whole cell patch clamp recording for the hippocampal cell in mice**


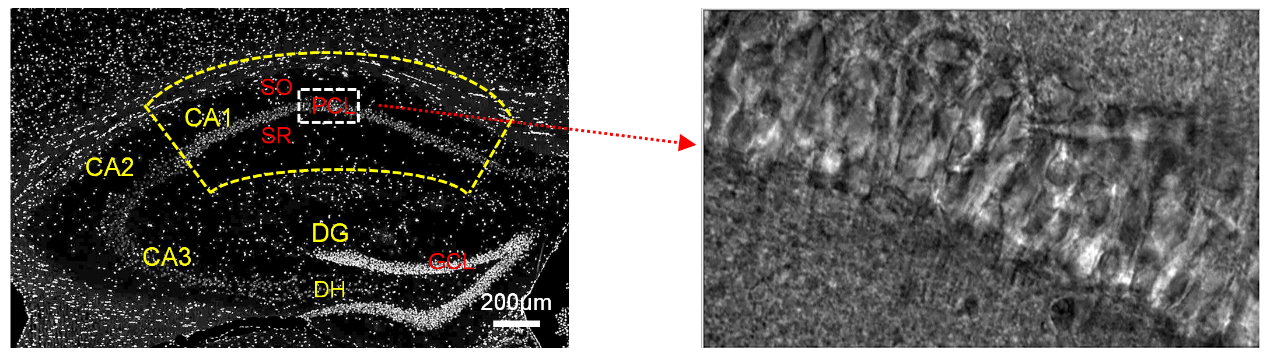


Anatomical diagram of the hippocampus (SO, stratum oriens; PCL, pyramidal cell layer; SR, stratum radiatum; DG, dentate gyrus; GCL, granular cell layer; DH, dentate hilus) (bar = 200 μm), and hippocampal CA1 pyramidal neurons are used for whole cell patch clamp recording.

**Figure S5. TEM imaging and detection of mitochondrial changes**


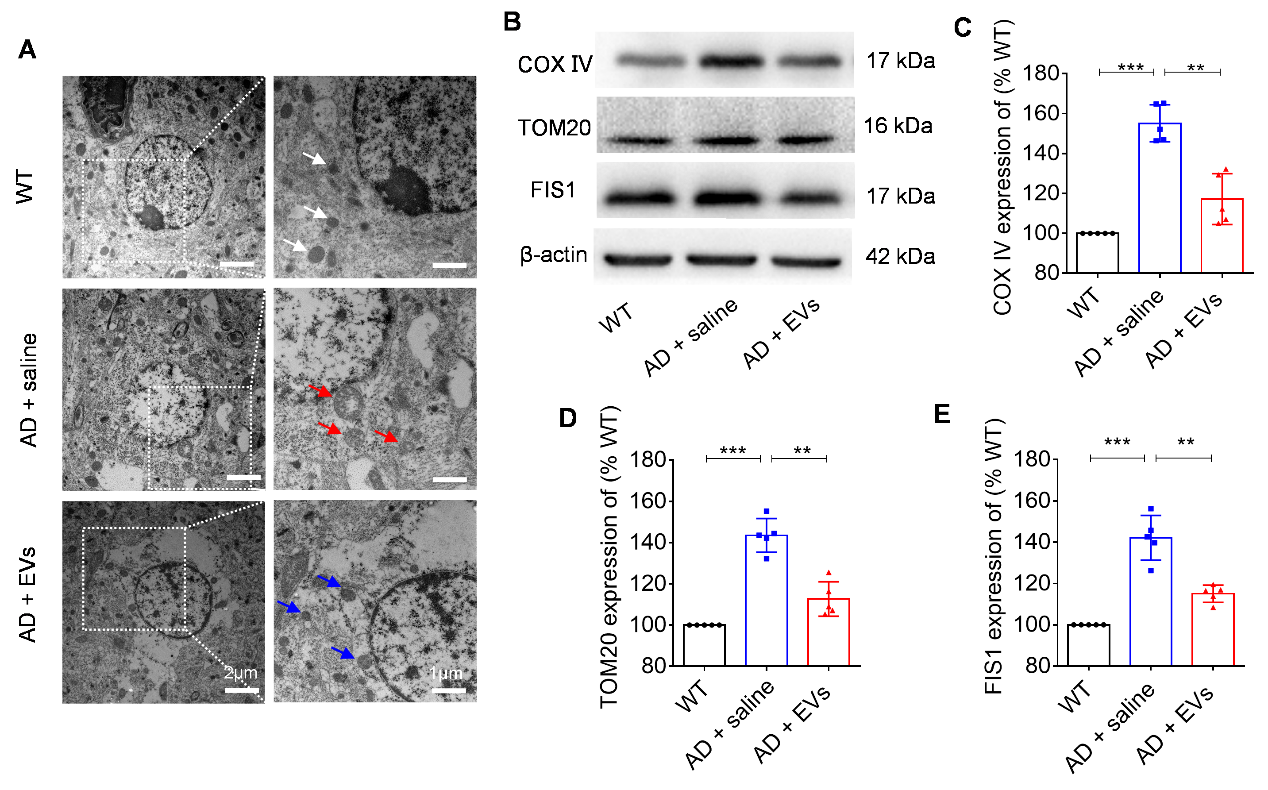


(A) Transmission electron microscopy (TEM) images of hippocampal CA1 pyramidal neurons in each group (bar = 2 μm), and on the right, higher magnification images (bar = 1 μm) display mitochondrial changes in the WT (white arrowheads), AD + saline (swelling and vacuolation, red arrowheads) and AD + EVs (blue arrowheads) groups. (B) Western blots of mitochondrial markers the relative expression of cytochrome c oxidase Ⅳ (COX Ⅳ), TOM20, mitochondrial fission protein 1 (FIS1) in the hippocampus of each group. (C-E) Histogram show the relative expression of COX Ⅳ (H), TOM20 (I) and FIS1 (J) after saline or MSC-EVs injection for 1 month (n = 5 per group). The values represent as Mean ± SEM. The data meet normal distribution and the variance is homogeneous. ^*^*P*<0.05, ^**^*P*<0.01, ^***^*P*<0.001, ^****^*P*<0.0001.

**Table S1. Short tandem repeat (STR) genomic profiling report**

**Typing results:**

| Loci | Cell line | | | Database | | |
| --- | --- | --- | --- | --- | --- | --- |
|  | Name：SH-SY5Y | | | Name：SH-SY5Y | | |
|  | Allele1 | Allele2 | Allele3 | Allele1 | Allele2 | Allele3 |
| D5S818 | 12 | 12 |  | 12 | 12 |  |
| D13S317 | 11 | 11 |  | 11 | 11 |  |
| D7S820 | 7 | 10 |  | 7 | 10 |  |
| D16S539 | 8 | 13 |  | 8 | 13 |  |
| VWA | 14 | 18 |  | 14 | 18 |  |
| TH01 | 7 | 10 |  | 7 | 10 |  |
| AMEL | X | X |  | X | X |  |
| TPOX | 8 | 11 |  | 8 | 11 |  |
| CSF1PO | 11 | 11 |  | 11 | 11 |  |
| D12S391 | 18 | 22 |  |  |  |  |
| FGA | 23.2 | 24 |  |  |  |  |
| D2S1338 | 17 | 19 |  |  |  |  |
| D21S11 | 31 | 31.2 |  |  |  |  |
| D18S51 | 13 | 16 |  |  |  |  |
| D8S1179 | 15 | 15 |  |  |  |  |
| D3S1358 | 15 | 16 |  |  |  |  |
| D6S1043 | 12 | 18 |  |  |  |  |
| PENTAE | 7 | 11 |  |  |  |  |
| D19S433 | 13 | 14 |  |  |  |  |
| PENTAD | 10 | 12 |  |  |  |  |


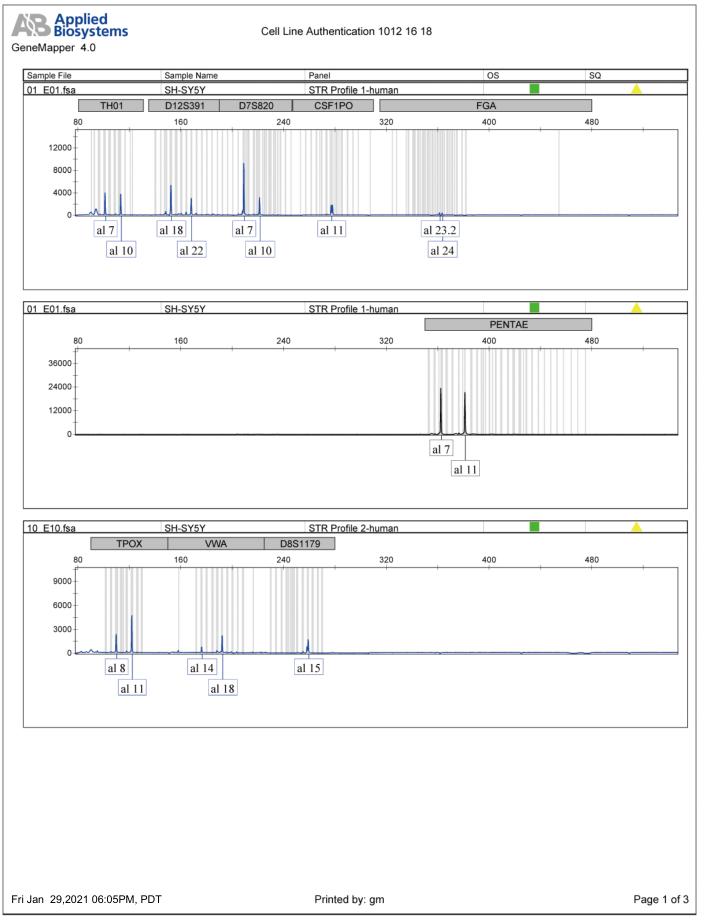
**Cell line phenotype：**


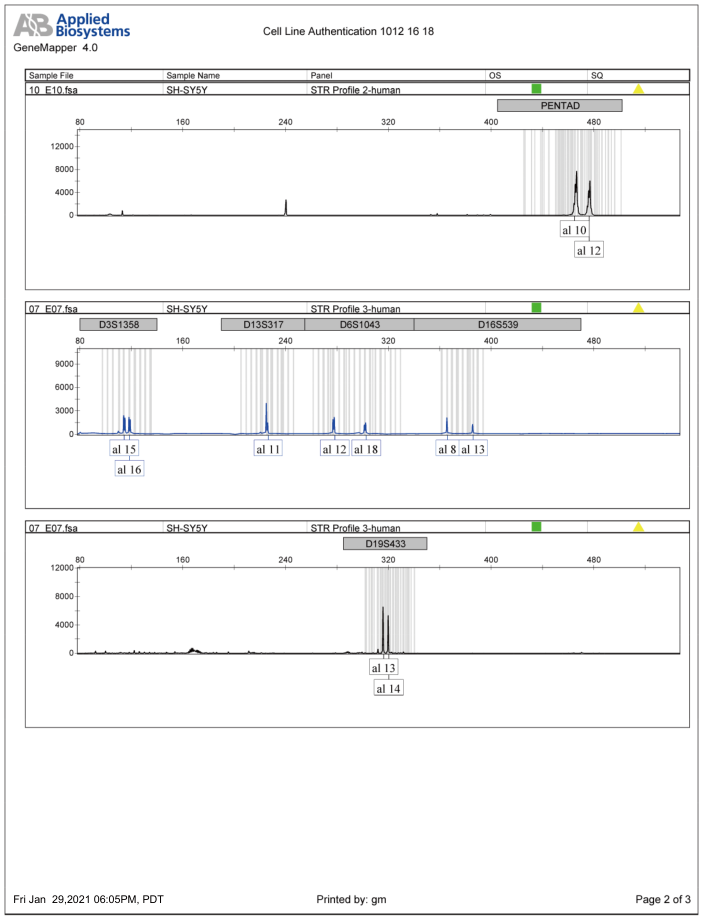

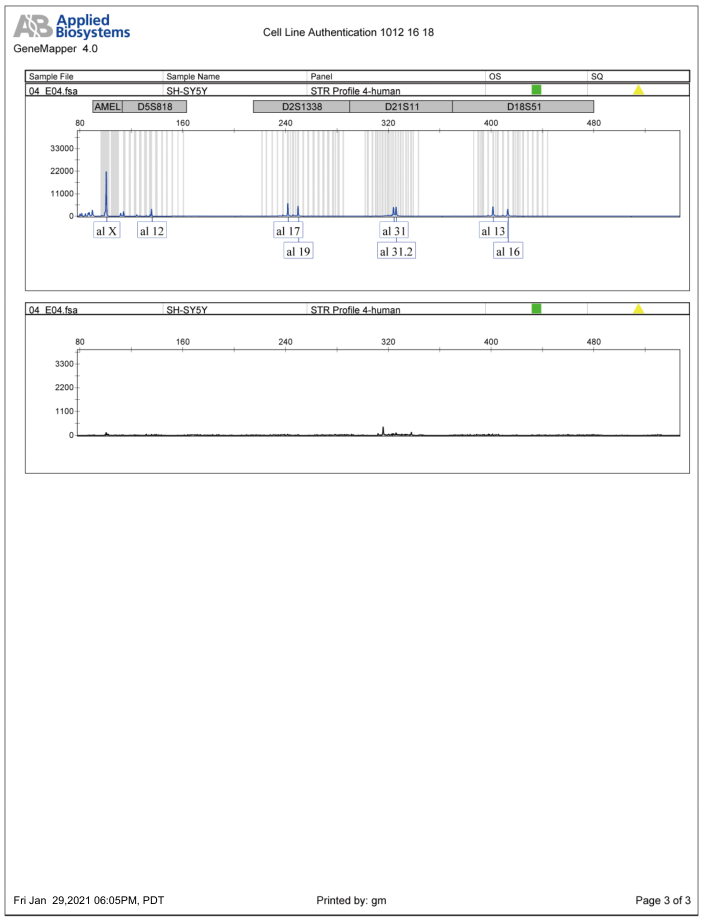

Supplement: Supplementary file 1 — Supplemental information [file 41420_2021_620_MOESM1_ESM.docx]
