# Supplementary material for: Tail-vein injection of MSC-derived small extracellular vesicles facilitates the restoration of hippocampal neuronal morphology and function in APP / PS1 mice
Source: Cell Death Discov. 2021 Sep 4;7:230. doi: 10.1038/s41420-021-00620-y (PMC8418600; doi:10.1038/s41420-021-00620-y)
Supplement: Supplementary file 2 — Dataset 1 [file 41420_2021_620_MOESM2_ESM.pdf]

DECLARATION OF CONTRIBUTIONS TO ARTICLE

ADMC

Manuscript Number:	Journal Name:	
CDDISCOVERY-21-1925-T	*Cell Death Discovery*	(the 'Journal')

Proposed Title of the Contribution:

Authors' Contribution for Cell Death Discovery (the 'Contribution)

Author(s):

Han Wang, Yuqi Liu, Junchen Li, Tian Wang, Yue Hei, Huiming Li, Xue Wang, Lina Wang, Ruijing Zhao, Weiping Liu, Qianfa Long (the 'Authors')

For all *CDDis*covery articles, each person named as an author in the published version must be able to show he or she has contributed substantially to the article.

Authorship credit should be based on 1) substantial contributions to conception and design, acquisition of data, or analysis and interpretation of data; 2) drafting the article or revising it critically for important intellectual content; and 3) final approval of the version to be published. Authors should meet conditions 1, 2 and 3.

Any person who cannot be shown to have made a substantial contribution to the article cannot be listed as an author in the final version. The name of any person who is deemed to have made a minor contribution can, however, appear in the Acknowledgments section of the article.

Please complete the table below to indicate the contributions of all named authors to the manuscript.

Author Full Name:	Specification of Contribution to the Manuscript:
Han Wang	HW performed the experiments
Yuqi Liu	YL performed the experiments
Junchen Li	JL interpreted the data
Tian Wang	TW performed the experiments
Yue Hei	YH interpreted the data
Huiming Li	HL performed the experiments
Xue Wang	XW performed the experiments
Lina Wang	LW performed the experiments
Ruijing Zhao	RZ performed the data analysis
Weiping Liu	WL designed experiments
Qianfa Long	QL designed experiments and wrote the manuscript.

Please complete the table below to indicate the contributions of all named authors to the figures.

Figure 1:

TW generated the data and prepared panel A-C, HW generated the immune-histochemistry data and labelled the image.

Figure 2:

JL generated the data and prepared panel B, D, F and H, WL generated the immune-histochemistry and Western blotting data.

Figure 3:

YL generated the data, JL prepared panel A-G.

Figure 4:

HW generated the immune-histochemistry data, TW and YH generated the data and prepared panel B-E.

Figure 5:

RZ generated the immune-histochemistry data (A), LW generated the Patch-clamp data (H), HL prepared panel B-G, I-K.

Figure 6:

XW generated the Weatern blotting data (in vivo), LW generated the Weatern blotting data (in vitro), QL prepared panel A-L.

Signed for and on behalf of the Author(s):	Print Name:	Date:
Qianfa Long	Qianfa Long	July 28, 2021
